# *Anais Brasileiros de Dermatologia*: 100 years of history (1925‒2025)^☆^

**DOI:** 10.1016/j.abd.2024.08.001

**Published:** 2024-11-05

**Authors:** Sílvio Alencar Marques, Ana Maria Ferreira Roselino, Hiram Larangeira de Almeida Junior, Luciana Patrícia Fernandes Abbade

**Affiliations:** a Department of Infectology, Dermatology, Diagnostic Imaging and Radiotherapy, Faculdade de Medicina, Universidade Estadual Paulista, Botucatu, SP, Brazil; b Department of Internal Medicine, Division of Dermatology, Faculdade de Medicina de Ribeirão Preto, Universidade de São Paulo, Ribeirão Preto, SP, Brazil; c Department of Dermatology, Universidade Federal de Pelotas, Pelotas, RS, Brazil; d Department of Dermatology, Universidade Católica de Pelotas, Pelotas, RS, Brazil

**Keywords:** *Anais Brasileiros de Dermatologia*, Dermatology, History

## Abstract

The month of January 1925 marks the birth of the *Annaes Brasileiros de Dermatologia e Syphilografia* and currently, 100 years later, with great merit, we celebrate its evolution into *Anais Brasileiros de Dermatologia* (ABD). Indeed, those few brilliant pioneers never dreamed of the strength of the Brazilian Society of Dermatology nowadays. However, perhaps they envisioned a relevant role for ABD, with a natural space for dermatoses of infectious etiology, always prevalent in tropical and subtropical regions. And thus, it was established for decades. Currently, Brazilian Dermatology and ABD are plural and open to the different facets of Dermatology. However, both the specialty, Dermatology, and its official body, ABD, value and pay homage to history, and cannot forget or stray from it. Numerous challenges have been faced over the past 100 years. Many other challenges still remain, but it is up to us, as a gift to the 100^th^ anniversary of the *Anais Brasileiros de Dermatologia*, to learn about them and reflect on them. The following text provides a summary of the history of ABD over the years. We invite national and international readers to celebrate with us.

## Foundation of the Brazilian Society of Dermatology and the *Bulletin* in 1912

**Figure 1:**
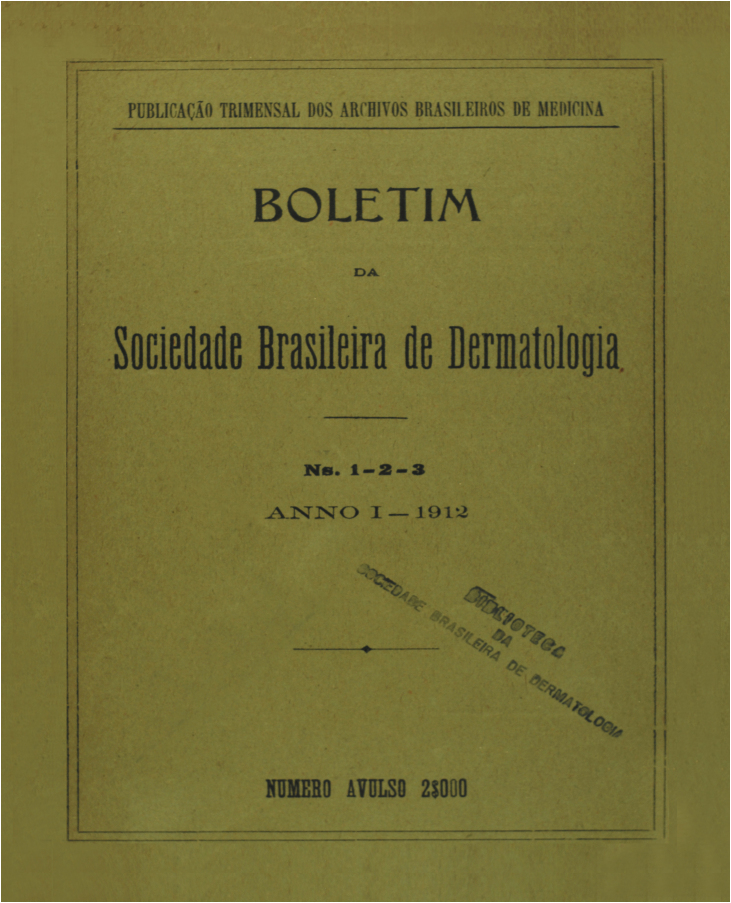
First Bulletin of the Brazilian Society of Dermatology ‒ 1912.

The history of *Anais Brasileiros de Dermatologia* (ABD) began in 1912 with the founding of the Brazilian Society of Dermatology (SBD, *Sociedade Brasileira de Dermatologia*) and, in the same year, the publication of the first Bulletin of the Brazilian Society of Dermatology (Fig. 1).^1^, ^2^ This publication aimed to disseminate clinical cases presented at scientific meetings held at the Santa Casa de Misericórdia in Rio de Janeiro, as well as to publish discussions arising from these meetings. The predominant topics in this first bulletin were infectious diseases, particularly leishmaniasis and blastomycosis (currently, paracoccidioidomycosis). Under the initial direction of dermatologist Dr. Fernando Terra, the Bulletin was published quarterly between 1912 and 1920 and it is considered a precursor and inspiration for ABD.

## *Annaes Brasileiros de Dermatologia e Syphilographia/Anais Brasileiros de Dermatologia e Sifilografia/Anais Brasileiros de Dermatologia* (1925 to nowadays)

**Figure 2:**
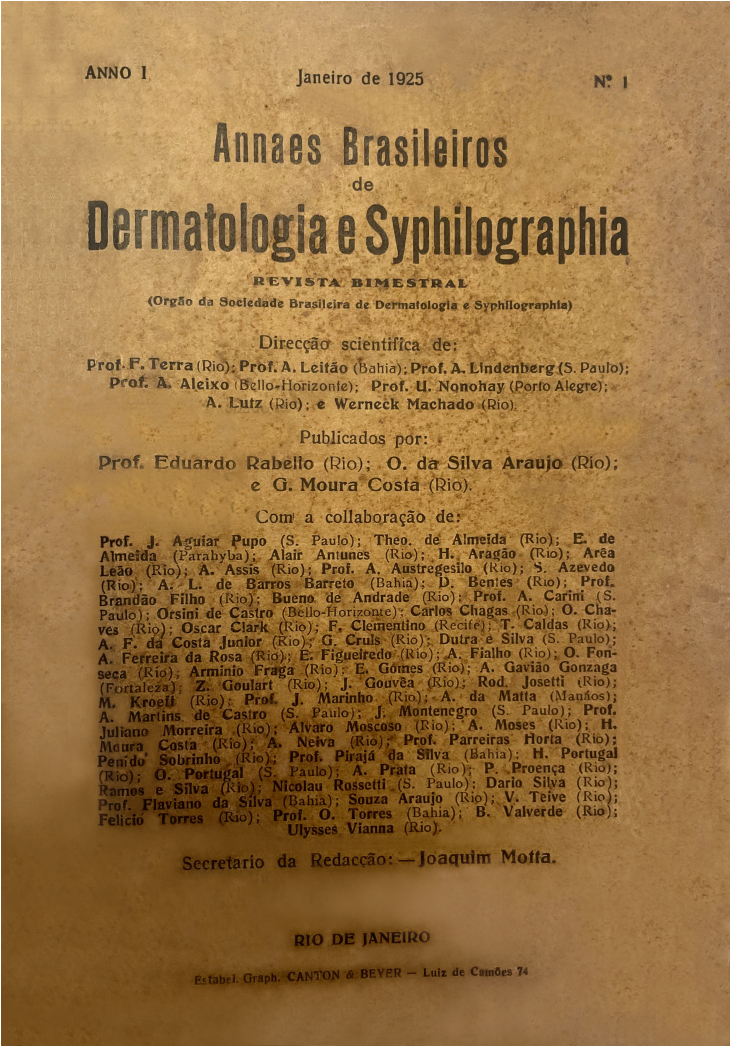
First issue of *Annaes Brasileiros de Dermatologia e Syphilografia* ‒ 1925.

**Figure 3:**
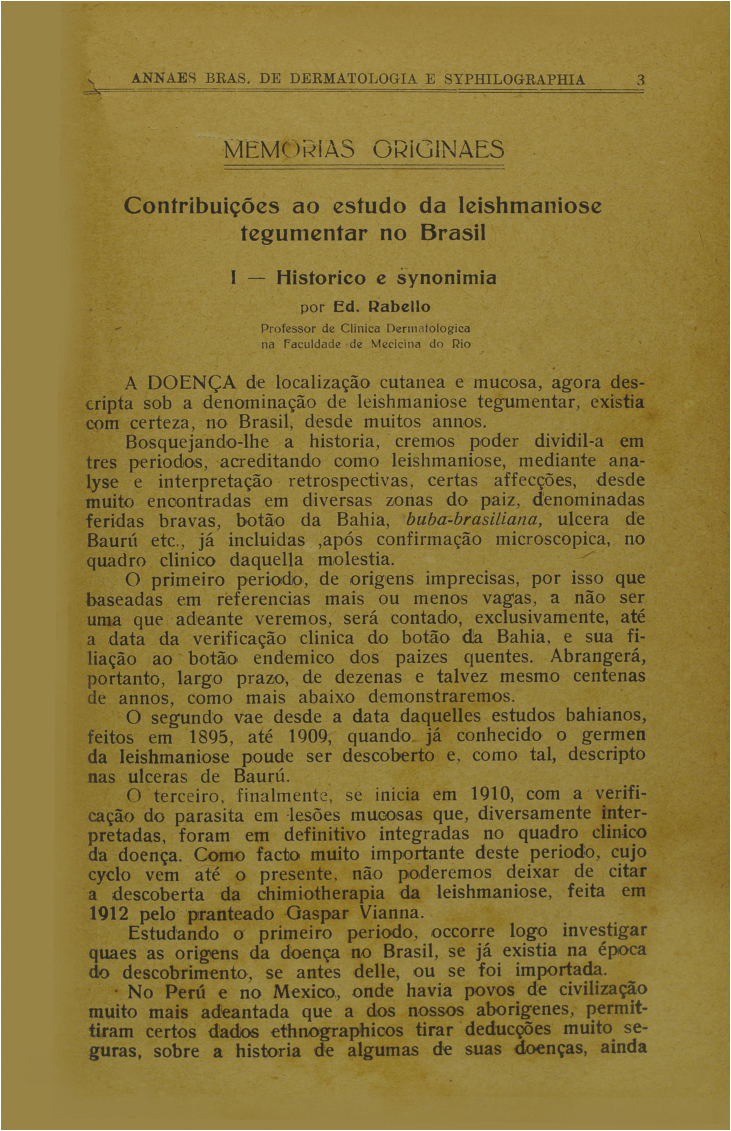
Original memoirs. Leishmaniasis.

ABD officially appeared in January 1925, through the hands of Prof. Eduardo Rabello, as Chief Editor, under the name *Annaes Brasileiros de Dermatologia e Syphilographia* (Fig. 2).^2^ The *Annaes*, in the editorial of its first issue, justified the initiative of creating a new scientific journal due to the growth of the specialty, the existence of Dermatology journals in other countries in South and North America, and the epidemiological relevance in Brazil of the then-called venereal diseases, particularly syphilis.^3^ Also, due to the increase of research into tropical diseases, including those of dermatological interest, initially under the leadership of the Instituto de Patologia Experimental de Manguinhos and, from 1917 onwards, under the definitive name of Instituto Oswaldo Cruz, both leadership centers for producing and stimulating scientific research. Just like the *Bulletin* that preceded them, the *Annaes* published clinical cases of interest, but it already incorporated scientific studies in the form of experimental laboratory tests and relevant conceptual articles as Original Memoirs (Fig. 3).^4^ Over the years, the *Annaes* overcame periods of national and international political crises, internal conflicts, external conflicts on a global scale, and economic difficulties of all kinds.

**Figure 4:**
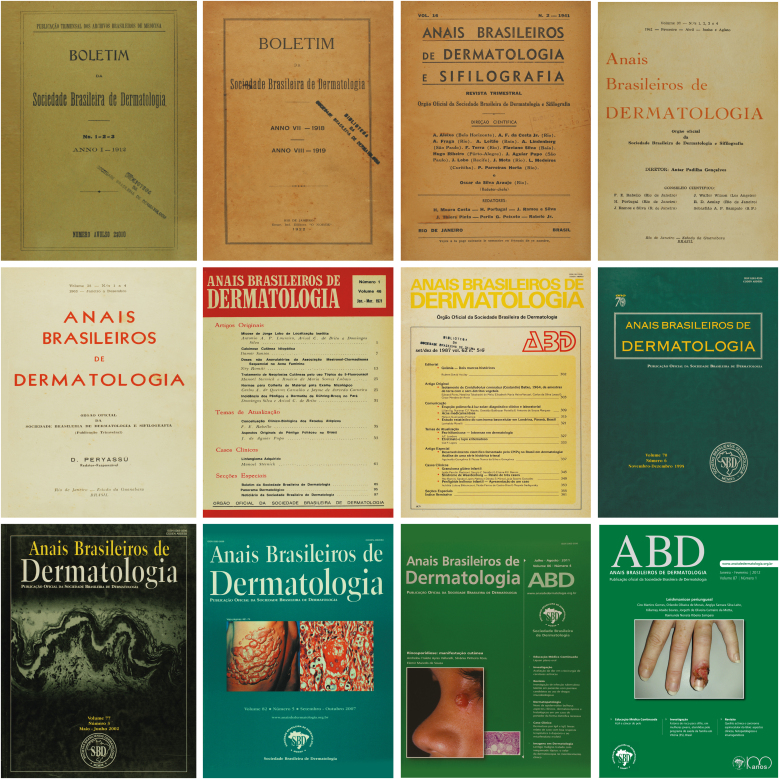
Historical covers (1912–2012).

The initial proposal of being a bimonthly journal was not always feasible, and during the year 1931, for purely economic reasons, the *Annaes* stopped being published and, in 1932, only one issue was published. From 1933 to the present day, the publication has been uninterrupted and it published three issues a year until 1983, when they were increased to six issues a year. The *Annaes* preceded the idea or the holding of the Brazilian Congresses of Dermatology and, therefore, since its founding, it has been one of the main transmitters of the academic word of SBD. At least during the first half of the 20^th^ century, due to the small number of Medical Schools, Dermatology Services, and Research Institutes with scientific production, ABD remained alive due to the dedication of a few dermatologists with an academic interest and mindset. Table 1 lists the Chief Editors of ABD from its foundation to the present day. Fig. 4 depicts different covers used over the years until they settled on the various shades of green. It should be noted that in 1933 the spelling of *Annaes Brasileiros de Dermatologia e Syphilographia* was changed to *Anais Brasileiros de Dermatologia e Sifilografia*, and in 1961 it was given its current and definitive name of *Anais Brasileiros de Dermatologia*. It is very important to note that there is no Brazilian Annals of Dermatology, as it is sometimes observed in articles or references.

**Table 1:** Chief Editors (1925‒2004). Scientific Editors (2004‒2025).

Name	Period
Eduardo Rabello	1925‒1926
Oscar da Silva Araújo	1927‒1937
Eduardo Rabello	1938‒1940
Oscar da Silva Araújo	1941
Francisco Eduardo Rabello	1942‒1945
Hildebrando Marcondes Portugal	1946‒1947
Antar Padilha-Gonçalves	1948‒1962
Demétrio Peryassu	1963‒1969
Rubem David Azulay	1970‒1972
Sílvio Fraga	1973
Rubem David Azulay	1974‒1992
Absalom Lima Filgueira	1993‒1997
Jesus Rodrigues Santamaría	1998
Leninha Valério do Nascimento	1998‒2004
Bernardo Gontijo	2004‒2008
Izelda Maria Carvalho Costa	2009‒2015
Sinésio Talhari	2016‒2020
Sílvio Alencar Marques	2021‒2025

## Achievement of indexing

A great leap in quality and modernity of ABD occurred from 1980 onwards and, even more so, from 1983 onwards, under the coordination of the then Chief Editor, Prof. Rubem David Azulay, when bimonthly publications were consolidated and an attempt was made to prioritize research articles.^5^ After being included in the LILACS (Latin American and Caribbean Literature in Health Sciences) database in 1981, ABD started being indexed in SciELO (Scientific Electronic Library Online) in 2003, when Prof. Leninha Valério do Nascimento held the position of Chief Editor (1998‒2004). It should be noted that efforts began, during this administration, to obtain indexing in PubMed, the search platform of the National Library of Medicine (NLM), which gathers records from the MEDLINE (Medical Literature Analysis and Retrieval System Online) database. This is the international reference database, implemented in 1997, and is extremely demanding and judicious in incorporating new journals. During this period, in addition to including new sections, with an emphasis on Continuing Medical Education, the first application for PubMed/MEDLINE indexing was made, which was unsuccessful for several reasons. However, it was a crucial step, since the reasons for the refusal were reported by the National Center for Biotechnology Information (NCBI), a division of the NLM in the United States of America. With the knowledge of these reports, the following administration (2004-2008), under Prof. Bernardo Gontijo as Chief Editor, began working to improve what had been identified as insufficient in the first application. This was clearly a difficult task since one of the assessed and criticized criteria was the low percentage of original research articles published in each issue. At the time, this situation of publishing few research articles was the result of ABD being mostly a national journal, with less power of attraction, in this regard, when compared to other national journals with greater status and tradition. In other words, the scientific production originating from postgraduate programs, research institutions, and Dermatology Services, as well as their most robust clinical cases, were directed to international journals or to the most prestigious national ones. Breaking the negative vicious cycle of publishing few research articles due to lack of status and, consequently, not attaining sufficient status due to the inability to publish a greater number of articles of higher scientific standard, was the main objective of the 2004-2008 management of ABD. During this administration, other initiatives were implemented, such as improved editorial and graphic techniques, greater care and demand regarding the quality of clinical and histopathological photographs, and the necessary rigor in complying with the periodicity required of high-quality journals. In 2008, confident in the good work performed, with care in providing detailed information regarding the revamped ABD, supported by the quality of the writing in the English language, a new request for indexing was submitted to NCBI/NLM.

**Figure 5:**
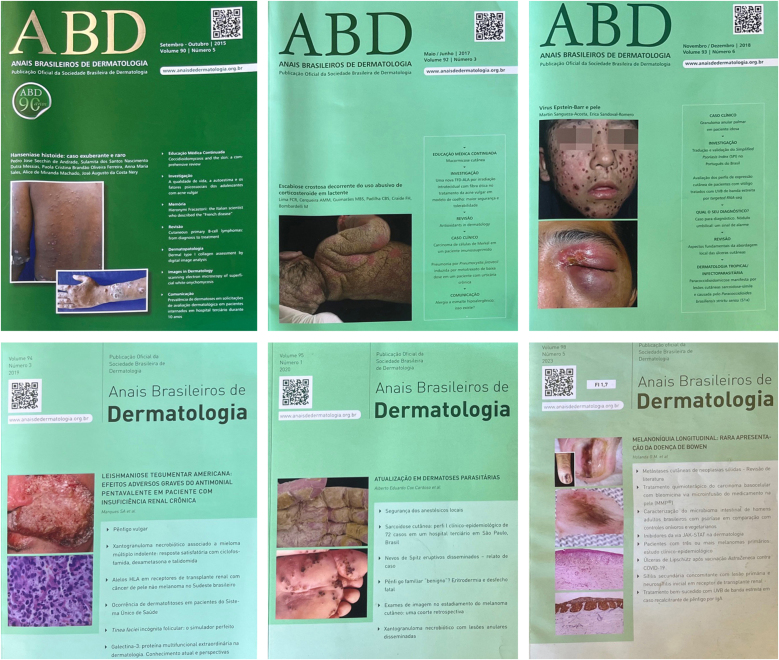
Historical covers (2013‒2024).

In 2009, during the following administration, under Prof. Izelda Maria Carvalho Costa (2009-2015), information was released that ABD had been formally accepted and was now included in the PubMed/MEDLINE database, an undisputed international reference. This achievement was a decisive one, constituting an essential milestone for ABD to grow and acquire the desired national and international status. Also during Prof. Izelda administration, ABD started having articles submitted to the journal and reviewed by reviewers in a completely digital, online format, which is also an essential step towards becoming a modern journal with international ambitions. Historical covers encompassing the period from 2012 to 2024 are shown in Fig. 5.

It is important to emphasize that, since its first issue, ABD has been fully funded by the Brazilian Society of Dermatology, characterizing itself as one of its academic voices. Equally commendable is the SBD respect for the academic independence of ABD. An additional notable characteristic is that it has remained, to this day, an open-access journal, in the Portuguese and English languages, with no submission or publication costs for neither national nor international authors.

## Growing metrics and international visibility

**Figure 6:**
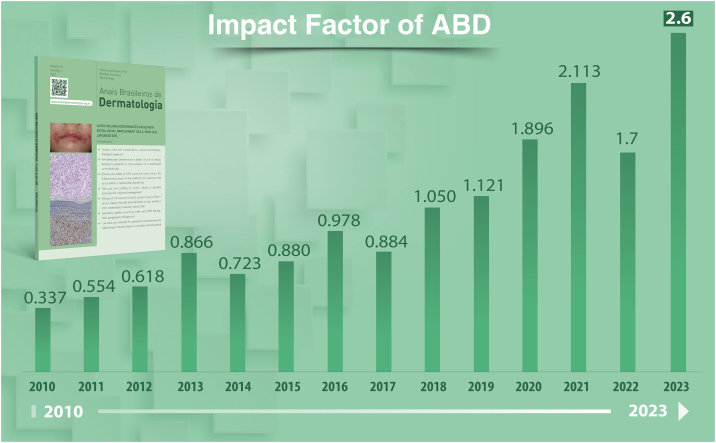
Impact factor of *Anais Brasileiros de Dermatologia* (2010‒2023).

Prof. Bernardo Gontijo requested to the Thomson-Reuters Corporation in 2008 that ABD be audited and in 2009 its first Impact Factor (IF) was reported and published by the Journal of Citation Reports. In 2010, ABD started with an IF = 0.337; in 2013, it was 0.866 and, 10 years later, in 2023, it had robust values ​​of IF = 2.6, occupying the 34^th^ position in the ranking of 91 journals of Dermatology and related specialties, that is, an evolution to Quartile 2 among the audited journals (Fig. 6).^6^ A decisive step towards this success must also be credited to the management of Chief Editor Prof. Sinésio Talhari (2016‒2020), transferring all the journal procedures to Elsevier Publishers, from the submission to final publication in print and online. This decision, which was not a simple one, had the financial and logistical support of the SBD Board at the time, with undeniable gains regarding the dissemination and consequent internationalization of the journal.

**Figure 7:**
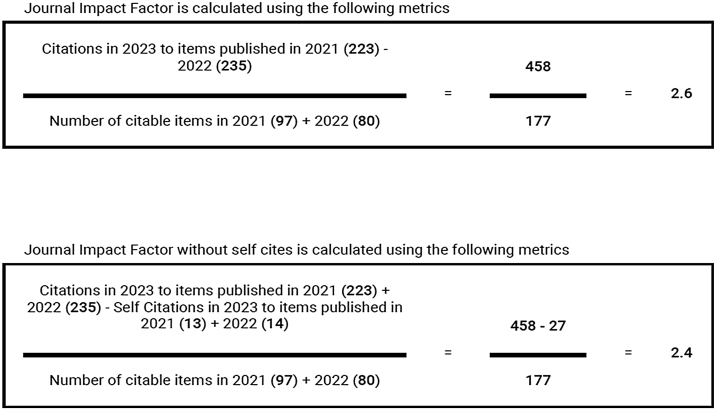
Calculation of the Impact Factor.

**Figure 8:**
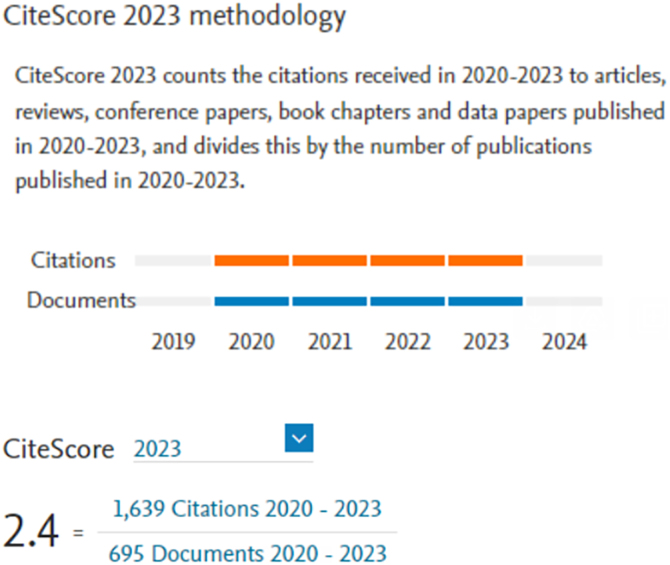
*CiteScore* calculation.

Another extremely important metric for scientific journals is CiteScore. While the IF counts citations received in the reference year for articles published in the two previous years and considers articles as citable according to its own criteria and a more restricted source journal database, CiteScore expands its analysis to include articles published in the four previous years. Furthermore, regardless of the section and type, all articles are counted to make up the denominator of the equation. The mathematical formulas used to arrive at the IF and CiteScore are shown in Figure 7, Figure 8, respectively.

Increasing national and international visibility and continuing to be an open-access journal with free publication resulted in a significant increase in the number of submissions. Consequently, it was necessary to implement a rigorous policy for selecting and accepting manuscripts, penalizing some of the sections most sought after by authors for submitting articles. The new reality and the need to maintain the growth dynamics of ABD have led the current management to gradually eliminate some sections and expand the “Letters” section, with subsections, some relevant to the interests of Dermatology at the time, such as “Letters-Therapy”, for example.^7^

## Challenges and proposals

*Anais Brasileiros de Dermatologia* and the *Actas Dermo-Sifiliográficas* are the only two journals from Ibero-Latin American countries indexed in the main scientific databases and, therefore, responsible for representing Ibero-Latin American Dermatology well. ABD are responsible for constantly updating and modernizing themselves, on par with journals with greater tradition and/or status. In this sense, the Deliberative Board of the Brazilian Society of Dermatology should discuss and decide on making the ABD published exclusively in English, aware that this is the international scientific language. We must be aware that even the Portuguese Society of Dermatology and Venereology has been publishing its official journal, the Portuguese Journal of Dermatology and Venereology, exclusively in English for several years. However, unlike the initiative of its sister society, the *sine qua non* proposal is to continue with the current and historical name of *Anais Brasileiros de Dermatologia*. Another proposal to be discussed will be whether or not the journal will remain in print or be published exclusively online. This is a somewhat controversial issue, but from an economic and sustainability point of view, it is an evolution that can be considered irreversible, since this initiative is already practiced by many journals, whether in Dermatology or related specialties. The continuation of the journal as an open-access journal should also be discussed, with or without maintaining full and free access, both in terms of submission and publication. Aiming to honor and respect the legal representation of SBD Deliberative Board, the decisions taken therein should reflect the will of all members and, for that reason, be accepted by all. Therefore, it is intended that when the year 2026 dawns, with new practices and also a new Chief Editor and, consequently, new management, ABD will aim at higher goals with greater clarity regarding the existing base and future challenges.

Last, but not least, ABD would like to express its immense gratitude to its Scientific Editors and members, over time, of the different Boards of Directors of the Brazilian Society of Dermatology, for their support, to the Authors, to the Reviewers, and to the daily and indispensable work of its Technical Collaborators.

## Financial support

None declared.

## Authors’ contributions

Silvio Alencar Marques: Approval of the final version of the manuscript; drafting and editing of the manuscript.

Ana Maria Roselino: Approval of the final version of the manuscript; drafting and editing of the manuscript.

Hiram Larangeira de Almeida Junior: Approval of the final version of the manuscript; drafting and editing of the manuscript.

Luciana P. Fernandes Abbade: Approval of the final version of the manuscript; drafting and editing of the manuscript.

## Conflicts of interest

None declared.
